# From Earth to orbit: cardiovascular innovation diffusion to space medicine - an updated review incorporating a novel predictive modeling framework

**DOI:** 10.3389/fphys.2026.1890835

**Published:** 2026-07-28

**Authors:** Fariba Yazdanpanah, Hamid Yazdanpanah Asmarz

**Affiliations:** 1 Department of General Preventive Medicine and Public Health, The University of Texas at Tyler, Tyler, TX, United States; 2 Center for Education and Research, Federal University of Santa Catarina, Florianópolis, Brazil

**Keywords:** aerospace medicine, cardiovascular health, diffusion of innovations, DOI, predictive modeling, preventive medicine

## Abstract

Extraterrestrial environments have an impact on cardiac output, vascular integrity, and endothelial function. This Update Review aims to integrate multi-domain cardiovascular preventive strategies through advanced biomedical and lifestyle interventions in long-duration spaceflight, as well as assessing the adoption of such innovations using Rogers’s Diffusion of Innovations (DOI) theory among spaceflight stakeholders. To conduct this Update Review, we restricted the inclusion criteria to conceptual and interdisciplinary literature in English related to spaceflight in the PubMed, Scopus, and Google Scholar datasets. Articles aligned with the study scope and the relevant keywords about spaceflight, cardiovascular health, and DOI were included. Thematic synthesis was used to integrate the results with the DOI theory and advanced prevention approach on cardiovascular health, with potential benefits in space medicine. Our assessment of current literature suggests that a combined, multi-domain framework for cardiovascular protection in extraterrestrial environments, incorporating lifestyle strategies with complementary biomedical approaches, would be most useful in the pre-Moon or Mars phase. This review underscores how strategic adoption of protective innovations, coupled with a communication model like DOI, can target late adopters and accelerate effective prevention strategies across the aerospace healthcare ecosystem.

## 1 Introduction

Cardiovascular health in space is affected by various factors, including immobility due to weightlessness, increased oxidative stress, and sympathetic nervous system activity, exposure to cosmic radiation, and disruption of the body’s circadian rhythm (Han et al., 2024; Sharma et al., 2023; Mendes Zambetta et al., 2024). In the microgravity environment of space, cardiac output, arterial compliance, and structural adaptation are reduced (Lee et al., 2020; Tuday et al., 2007; Pantalos et al., 1998) cosmic radiation and oxidative damage can also contribute to endothelial dysfunction; in addition, immobility and altered light cycles disrupt stress and metabolic regulation (Patel, 2020; Locatelli et al., 2022).

Historically, space medicine has emphasized strategies that are reactive to the extraterrestrial environmental conditions. However, growing evidence on Earth supports proactive preventative strategies that include corrective biomedical technologies and behavioral interventions to preserve cardiovascular function before pathology develops. Given the accelerated cardiovascular deterioration in microgravity, such preventive approaches may be even more critical in spaceflight and could be adopted to maintain astronaut health. In this Update Review, we hypothesize that cardiovascular protection in spaceflight may be optimized through a tiered approach ranging from (A) lifestyle-based prevention, (B) radiation protection countermeasures, (C) mitochondrial and endothelial therapies, (D) AI-aided diagnostics and wearable technologies, to (E) epigenetic and regenerative therapies (Han et al., 2024; Khan et al., 2026; Kim et al., 2016; Chambers et al., 2022). Furthermore, we propose that by providing a roadmap for collaboration between medical and space science stakeholders using the “Diffusion of Innovations” (DOI) framework (Rogers, 2003) for communication, the adoption and effective implementation of these innovations can be accelerated. The DOI theory/model was first proposed by Everett Rogers in 1962 and explains *how*, *why*, and at *what* rate (how quickly) new ideas and innovations spread within a population or social system.

Rogers’s DOI model classifies types of adopters based on their propensity to adopt innovations early or late, and describes the social dynamics through which new ideas diffuse (Rogers, 2003).

## 2 Methods

### 2.1 Review design

This study used a conceptual and theoretical review methodology to assess developments in cardiovascular countermeasures within the context of space medicine. Unlike empirical reviews, which synthesize findings from original data-driven studies such as clinical trials, cohort studies, or observational analyses, conceptual reviews integrate and interpret existing theoretical frameworks, models, and interdisciplinary literature to generate new hypotheses and conceptual frameworks. This approach is intended to support critical analysis of emerging models, current trends, and future directions in extraterrestrial health research.

### 2.2 Literature search strategy

A structured literature search was conducted in PubMed, Scopus, and Google Scholar. The search strategy combined controlled vocabulary and free-text terms using Boolean operators (AND/OR). The full search string included:

(“spaceflight” OR “space medicine” OR “aerospace medicine” OR “microgravity”) AND.

(“cardiovascular” OR “heart” OR “vascular function” OR “endothelial dysfunction”) AND.

(“prevention” OR “countermeasures” OR “exercise” OR “radiation” OR “oxidative stress”) AND (“innovation” OR “diffusion of innovations” OR “Rogers” OR “DOI theory” OR “technology adoption” OR “implementation science”).

Keywords were selected to capture three domains: (1) space physiology, (2) cardiovascular prevention, and (3) innovation adoption framework.

### 2.3 Inclusion and exclusion criteria

The sources used were relevant theoretical papers, conceptual models, interdisciplinary manuscripts, and books published in English between 1962 and 2025. In total, thirty-one published sources were screened and included. Research that was not supported by conceptual analysis was excluded unless it was considered theoretically useful. The DOI framework was not used as an inclusion criterion. Instead, it was applied *post hoc* as an interpretive and conceptual synthesis tool to map and categorize findings from the included literature. Therefore, studies were included based on thematic relevance to cardiovascular space medicine, innovation, and preventive strategies, regardless of whether DOI was explicitly referenced in the original publications.

### 2.4 Analytic structure

Thematic analysis and synthesis were conducted to obtain key abstract themes and align them with relevant models such as the DOI theory, models of primordial to tertiary prevention, and systems thinking for cardiovascular health in space travel. Concepts for the pathway were mapped and organized based on existing cardiovascular health strategies, which could be categorized into innovation and adoption in Rogers’s DOI model. In this framework, DOI model was applied *post hoc* as an interpretive tool to organize and categorize extracted themes rather than as a criterion for study inclusion.

### 2.5 Conceptual framework integration

This Update Review led to the development and addition of a concept map that illustrates how new and advanced interventions, such as epigenetic and regenerative therapies, combined with Artificial Intelligence(AI)-based biosensors, mitochondrial antioxidants, and circadian rhythm regulation, alongside innovation diffusion stages and levels of cardiovascular risk reduction in the pre-spaceflight phase, can help maintain heart health during extraterresterial travel.

## 3 Results

### 3.1 Proposed innovative strategies for promoting cardiovascular health in space missions

Spaceflight places profound stress on cardiovascular homeostasis due to microgravity, increased radiation exposure, and psychophysiological changes resulting from environmental unfamiliarity, feelings of isolation, and confinement (Baran et al., 2021; Marazziti et al., 2022). To mitigate these challenges, this Update Review suggests five innovative cardiovascular domains from the current medical literature to promote heart health resilience in space, as follows: A) Lifestyle-based prevention: Comprehensive in-flight measures, including daily in-flight exercise, chrono-nutrition, restorative sleep, and polyphenol-rich diets that can improve vascular flexibility and autoregulation (Petersen et al., 2016; Almoosawi et al., 2016; St-Onge et al., 2016; Najjar et al., 2021). B) Radiation-protection countermeasures: Heart tissue is particularly vulnerable to the effects of radiation during spaceflight. Although cosmic rays differ from ionizing radiation, deep space travel involves traveling through environments with inherent hazards of ionizing radiation, which can cause cellular injury by producing reactive oxygen species. Therefore, the application of radioprotectants against free radicals and oxidative stress, such as low-dose melatonin (5 mg/kg) and Tempol (20 mg/kg/day for 14 days), could also help alleviate DNA damage, apoptotic signaling, and endothelial dysfunction caused by cosmic radiation exposure (Patel, 2020; Demir et al., 2025; Tobeiha et al., 2022; Saheera et al., 2019). C) Mitochondrial and endothelial treatments: Since space-induced oxidative stress can also cause damage to mitochondria and the endothelium, preliminary research has shown that advanced therapies such as the mitochondrial-targeted peptide SS-31 (Elamipretide) in a mouse model and Nicotinamide Adenine Dinucleotide (NAD+) boosters, which act as oxidative stress reducers in humans (Chiao et al., 2020; Bhasin et al., 2023), could be considered as potential future preventive and therapeutic strategies to improve endothelial integrity and function, and vascular tone, in space. D) Artificial intelligence (AI) - aided diagnostics and wearables: AI technology with biosensors is still in its infancy; however, continuous biosensors and AI-enhanced wearables are capable of collecting a wealth of sensitive data and, by employing real-time physiological tracking, provide predictive analytics on various aspects of health-related metrics during space missions, including heart rate, blood pressure, oxygen saturation, skin temperature, level of physical activity, sleep patterns, biochemical markers (e.g., glucose, cortisol, lactate, electrolytes, and pH), in addition to environmental factors and location information (Shajari et al., 2023) for further analysis. E) Epigenetic and regenerative therapies: Emerging technologies such as Clustered Regularly Interspaced Palindromic Repeats (CRISPR)/Cas9-mediated gene modulation, nanotechnology-enhanced stem cell therapies, and gene/cellular reprogramming (Bonowicz et al., 2025; Sarathkumar et al., 2021) represent highly experimental approaches. While these strategies demonstrate promising preclinical potential for cardiac tissue protection and regeneration, their application in space medicine remains strictly theoretical at present. Importantly, their potential implementation would require rigorous evaluation of the benefit-risk balance, including long-term safety, off-target genetic effects, immunological risks, and ethical considerations. At this stage, such interventions should be considered exploratory and not operational countermeasures for human spaceflight. Their role is therefore limited to future translational research frameworks rather than near-term clinical application.

### 3.2 Adopting cardiovascular preventive strategies in spaceflight, through the DOI framework

This section examines the practical adoption of preventive innovation strategies targeting cardiovascular health during long-duration space missions, using the DOI theory/model as an analytical framework (Rogers, 2003). The DOI model categorizes types of adopters and describes the sequential stages of innovation diffusion. In the DOI model, the five key stages of adoption, also called “adoption stages” or “process stages,” describe *what* happens in the individual or organization’s decision process, while “adopter categories” describe *who* adopts and *when* they adopt the innovation compared to others. Understanding the relationship between process stages (Knowledge, Persuasion, Decision, Implementation, and Confirmation*)* and the adopter categories (Innovators, Early Adopters, Early Majority, Late Majority, and Laggards) is key to effectively applying the DOI framework. The following sections provide further insight into these two dimensions of DOI theory: i) five key process stages of the innovation adoption: these describe the stages that an individual or organization goes through when faced with innovation: *Knowledge:* understanding how the innovation works. *Persuasion:* forming an attitude or opinion, which can be favorable or unfavorable. *Decision:* choosing between accepting and rejecting the innovation. *Implementation:* putting innovation into regular use. *Confirmation:* reinforcing the decision through results or aborting it if the results are not satisfactory (Rogers, 2003; Greenhalgh et al., 2004). **Table 1** describes these process stages and provides a summary of the application of these five stages of the DOI model to promote cardiovascular health in spaceflight. ii) adopter categories for innovation uptake: according to Rogers’s DOI theory (1962), adopter categories are groups of individuals or organizations that have different attitudes and levels of risk-taking, which affect *how quickly* they tend to adopt innovations. The adopter groups form a bell-shaped curve, often known as the “adoption curve” or “diffusion of ideas”, and are divided into discrete adopter groups as follows (Rogers, 2003; By Rogers Everett - Based on Rogers, E. (1962)): *Innovators* (2.5%): refers to the very first people to try an innovation. “Risk takers”. *Early Adopters* (13.5%): This category comprises respected opinion leaders who adopt an innovation early but more cautiously than innovators. *Early Majority* (34%): This group includes adopters who take their time to consider the benefits before accepting an innovation. *Late Majority* (34%): This set includes skeptical and conservative adopters, who only accept an innovation after the majority has accepted it. *Laggards* (16%): “The last to adopt”; often resistant to change, and habitually rooted in tradition. As shown in **Table 2**, the five adopter categories (Innovators, Early Adopters, Early Majority, Late Majority, and Laggards) have their own characteristics and probabilities in terms of the adoption of certain innovation strategies for cardiovascular health, depending on the level of readiness and acceptance of new technologies by the stakeholders of spaceflight. Innovators, characterized by their willingness to take risks and embrace emerging biotechnologies, are expected to be the first to adopt cutting-edge solutions such as epigenetic/regenerative therapies, AI-aided diagnostics, and wearable monitoring devices. According to Rogers’s adoption curve, only 2.5% of members of a social system fall in this category. Early Adopters, who are typically respected thought leaders, and account for 13.5% of the adoption curve, may follow suit by integrating AI-aided diagnostics and wearable, mitochondrial, and endothelial treatments, and radiation protection countermeasures. As clinical evidence accumulates, the next group of adopters, the Early Majority, a pragmatic, evidence-seeking group, will likely implement these innovations, particularly in the form of standard treatments and prevention protocols, once approved. The Late Majority, who are often more conservative and skeptical, may adopt novel measures only after they are fully established as routine practices, as well as at the level of lower-risk innovations, such as radiation protection countermeasures or lifestyle-based prevention. The share of each of the Early Majority and Late Majority groups on the Adoption Curve is 34%. Finally, Laggards, who prefer traditional methods and resist change, are likely to accept only minimal interventions, such as lifestyle-based prevention strategies, and only after large-scale normalization; 16% of the social system falls into the Laggards adoption category. Taken together, Sections 3.1. and 3.2. underscores that promoting cardiovascular resilience in long-duration spaceflight requires not only access to innovation and advanced technologies, but also strategic and systematic dissemination throughout the spaceflight ecosystem. Successfully transforming emerging innovation into operational space medicine, as conceptualized within the DOI framework, depends on aligning scientific readiness with stakeholder acceptance dynamics.

**Table 1:** Proposed application of the Diffusion of Innovations (DOI) process stages to cardiovascular health promotion in spaceflight.

Process stage (Rogers, 2003)	Description (Rogers, 2003)	Application to cardiovascular health promotion in spaceflight (Rogers, 2003; Petersen et al., 2016; Almoosawiet al., 2016; St-Onge et al., 2016; Najjar et al.,2021; Demir et al., 2025; Tobeiha et al., 2022;Saheera et al., 2019; Chiao et al., 2020; Bhasin etal., 2023; Shajari et al., 2023; Bonowicz et al.,2025; Sarathkumar et al., 2021; Greenhalgh et al.,2004)
Knowledge	Awareness of innovation and understanding how it works	Educate astronauts and the aerospace medicine team about oxidative stress treatments, AI^⁑^ diagnostics, cosmic radiation countermeasures, lifestyle modifications, epigenetic/regenerative therapies.
Persuasion	Forming an attitude or opinion (favorable vs. unfavorable)	Share evidence from space analog studies, Early trials and expert endorsements.
Decision	Choosing to accept or reject the innovation	Support mission planners and astronauts in informed decision-making on new devices or therapies to protect heart health.
Implementation	Putting innovation into regular use	Provide training, protocols, and technical support for the in-flight use of diagnostics and therapeutics.
Confirmation	Reinforcing vs. aborting the decision through observed outcomes	Monitor outcomes and provide feedback loops to validate and sustain the use of innovation in spaceflight environments.

⁑ Artificial intelligence.

Innovation process stages and descriptions adapted from Rogers E. Diffusion of Innovations. 5th ed.2003 (Rogers, 2003). The last column (Application to Cardiovascular Health Promotion in Spaceflight) is the original synthesis by the authors.

**Table 2:** The likelihood of adopting innovative cardiovascular health strategies in spaceflight based on the Diffusion of Innovations (DOI) framework.

Adopter category	Adopter’s characteristics	Potential innovation strategies for cardiovascular health in spaceflight
Innovators	Risk takers, technology enthusiasts, first to try new ideas	Epigenetic/regenerative therapies (E) **^a^**, early AI^b^ - aided diagnostics, and wearable devices (D) **^c^** (Shajari et al., 2023; Bonowicz et al., 2025; Sarathkumar et al., 2021)
Early Adopters	Opinion leaders, respected experts	AI^b^- aided diagnostics, and wearable devices (D) **^c^**, mitochondrial and endothelial therapies (C) **^d^**, radiation-protection countermeasures (B) **^e^** (Demir et al., 2025; Tobeiha et al., 2022; Saheera et al., 2019; Chiao et al., 2020; Bhasin et al., 2023; Shajari et al., 2023)
Early Majority	Pragmatic, evidence-seeking	Mitochondrial and endothelial therapeutics after clinical validation (C) **^d^**, radiation-protection countermeasures (B) **^e^** as standard practice, lifestyle-based prevention (A) **^f^** (Petersen et al., 2016; Almoosawi et al., 2016; St-Onge et al., 2016; Najjar et al., 2021; Demir et al., 2025; Tobeiha et al., 2022; Saheera et al., 2019; Chiao et al., 2020; Bhasin et al., 2023)
Late Majority	Skeptical and conservative	Radiation-protection countermeasures (B) **^e^** as standard practice, routine adoption of lifestyle-based prevention (A) **^f^** (Petersen et al., 2016; Almoosawi et al., 2016; St-Onge et al., 2016; Najjar et al., 2021; Demir et al., 2025; Tobeiha et al., 2022; Saheera et al., 2019)
Laggards	Resistant, prefer traditional protocols	Minimal intervention approaches such as lifestyle-based prevention (A) **^f^** (Petersen et al., 2016; Almoosawi et al., 2016; St-Onge et al., 2016; Najjar et al., 2021)

**a, b, c, d, e, f** The letters A, B, C, D, and E refer to the proposed domains of innovation in the field of promoting cardiovascular health in space missions, which are mentioned in Section 3.1 under the title of Results.

b Artificial intelligence.

Adopters’ category and the characteristics of each adopter are taken from Rogers E. Diffusion of Innovations. 5th ed. 2003 (Rogers, 2003). The last column (Potential Innovation Strategies for Cardiovascular Health in Spaceflight) is originally developed by the authors.

## 4 Discussion

**Figure 1:**
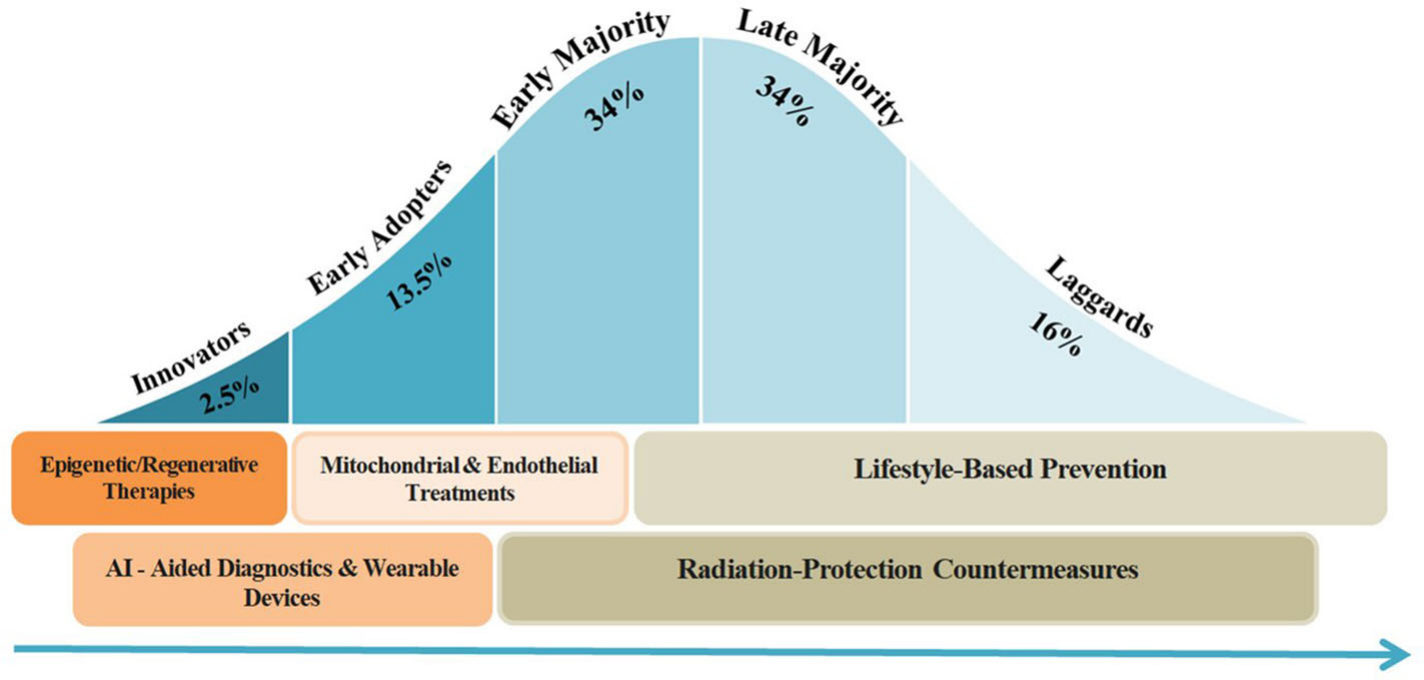
Proposed conceptual model integrating innovation strategies with Rogers Diffusion of Innovations (DOI) theory for cardiovascular health promotion in spaceflight. The bell-shaped curve content is derived from the Rogers’s adoption curve (Rogers, 2003). The model illustrating the application of potential innovative strategies for cardiovascular health promotion during spaceflight is shown below the bell-shaped curve and was designed by the authors.

This Update Review sheds light on the challenges of cardiovascular health during spaceflight and proposes a structured framework for adopting innovation guided by Rogers’s DOI theory. Consistent with previous investigations, microgravity, cosmic radiation, oxidative stress, and disruption of circadian rhythm collectively impair cardiovascular hemostasis in space travelers (Han et al., 2024; Sharma et al., 2023; Mendes Zambetta et al., 2024; Lee et al., 2020; Patel, 2020; Marazziti et al., 2022). However, unlike previous studies that have emphasized various pathophysiological mechanisms and conventional countermeasures in space travel (Tuday et al., 2007; Pantalos et al., 1998), this review aimes to shift the ground from reactive countermeasures to proactive, and innovation-based model for protecting cardiovascular health in space and enrich the discourse by incorporating advanced biomedical technologies such as mitochondrial-targeted peptide SS-31, NAD+ boosters, AI-based diagnostics, wearable monitoring technologies, and epigenic/regenerative therapies into a structured translational pathway in space medicine (Petersen et al., 2016; Almoosawi et al., 2016; St-Onge et al., 2016; Najjar et al., 2021; Demir et al., 2025; Tobeiha et al., 2022; Saheera et al., 2019; Chiao et al., 2020; Bhasin et al., 2023; Shajari et al., 2023; Bonowicz et al., 2025; Sarathkumar et al., 2021). Furthermore, our approach leverages the DOI theory as a conceptual tool to map variation in stakeholder readiness and adoption rates across the spaceflight ecosystem to highlight the critical role of communication and collaboration between medical teams and space agencies in implementing innovations. Although Rogers’s DOI model is often used in the healthcare setting, we first applied it to space medicine to provide a framework for setting communication strategies, aligning risk tolerance, and facilitating phased implantation of proposed cardiovascular innovations in the space medicine context. As **Figure 1** illustrates, our depicted diffusion framework categorizes heart health innovations into domains ranging from lifestyle-based modifications to advanced biomedical technologies, thereby reducing the heterogeneity, simplifying complexity, and supporting the strategic deployment of innovations among diverse stakeholders in the space industry. This perspective complements existing research on cardiovascular health in space by underscoring the social and organizational dynamics that influence technology uptake beyond mere efficacy and safety considerations. In doing so, our review responds to ongoing calls for multimodal preventive strategies (Khan et al., 2026; Kim et al., 2016) while also emphasizing the importance of phased adoption pathways. We believe aligning technological innovation with operational healthcare systems is essential for long-term missions, particularly as emerging therapies transition from experimental stages to potential practical applications. For instance, mitochondrial or NAD+ related therapeutic strategies have shown promise in early human studies and animal models in aging biology, reversing cardiac dysfunction and improving cardiac function (Chiao et al., 2020; Bhasin et al., 2020), so they will also have a potential positive impact on cardiac health in extratresterial environments. However, their integration into operational space missions requires structured dissemination of evidence, rigorous validation protocols, and alignment with space mission-specific safety standards. Similarly, combining AI-based wearable sensors with health technologies may enable continuous monitoring of health parameters, early detection of dysfunction, and adaptive care intervention in space environments. Such an approach can support the broader vision of P4 medicine (Predictive, Preventive, Personalized, Participatory) and create a scalable platform for preventive, diagnostic, therapeutic, and follow-up care across various healthcare domains, including space medicine (Shajari et al., 2023; Johnson et al., 2021). Importantly, this Update Review also emphasizes the relationship between technology readiness and stakeholder risk tolerance, a dimension that is often under-explored in space health planning for the timely application of innovation to control health risks. As noted in a previous systematic review of service organizations by Greenhalgh et al., conservative operational stakeholders often exhibit resistance to innovation and change (Greenhalgh et al., 2004). Building on this insight, our DOI-based framework suggests customized engagement strategies that target specific categories of adopters (Early Majority, Late Majority, and Laggards), who need greater communication and awareness about technological advances to increase their adoption motivations. By anticipating barriers to diffusion and aligning innovation strategies with stakeholder profiles, this framework provides a practical roadmap for accelerating the transition of cardiovascular health interventions from conceptual development to routine operational use as a standard, not the exception. Together, these elements contribute to a more comprehensive understanding of how to responsibly and efficiently integrate modern lifestyle pillars and novel biomedical innovations into long-duration space missions, so that adverse cardiovascular outcomes in extraterrestrial environments are minimized. However, this Update Review is limited by its reliance on a conceptual context and theoretical modeling derived from secondary data. Empirical validation through controlled experimental trials, cohort studies, and space mission-based implantation research is needed. We recommend future research to examine how real-time feedback from wearable sensors, in combination with biomedical and behavioral interventions, impacts astronaut health outcomes. In addition, investigations should assess whether the diffusion patterns observed in terrestrial healthcare environments translate to the unique operational and cultural dynamics of space and its crews (Majumder et al., 2017; Vakoch, 2011).

## 5 Conclusion

Summing up, this Update Review is a bridge between cutting-edge medical innovations and organizational communication theories, creating a comprehensive understanding of how to optimally collaborate and implement cardiovascular health solutions in the space flight industry. Such a model could be implemented in the pre- Moon and Mars phase to increase the safety of space crews as humanity prepares for longer and even more challenging journeys to survive beyond our home planet, Earth.

## Data Availability

The original contributions presented in this study are included in the article. Further inquiries can be directed to the corresponding author.
